# Supplementary material for: Taming the perils of photosynthesis by eukaryotes: constraints on endosymbiotic evolution in aquatic ecosystems
Source: Commun Biol. 2023 Nov 11;6:1150. doi: 10.1038/s42003-023-05544-0 (PMC10640588; doi:10.1038/s42003-023-05544-0)
Supplement: Supplementary file 2 — Supplementary Table 1 [file 42003_2023_5544_MOESM2_ESM.pdf]

Supplementary Table 1. Mechanisms to mitigate photosynthetic oxidative stress evolved in eukaryotes other than ROS scavenging.

Mechanisms	Notes
**(Algae and plants)**	Reviewed in refs.[7,8,12].
Host movement depending on the light intensity	
Relocating chloroplasts depending on the light intensity	
Shading/sunscreening under high light	
Digesting dysfunctional chloroplasts (in plants)	Reviewed in ref.[62].
Maintenance and regulation of the photosynthetic apparatus by the host genome	
**(*P. chromatophora*)**	Ref.[67].
Maintenance and regulation of the photosynthetic apparatus by the host genome	

Supplementary Table 1. Mechanisms to mitigate photosynthetic oxidative stress evolved in eukaryotes other than ROS scavenging (*continued*).

Mechanisms	Notes
**(Kleptoplasty)**	
Shading/sunscreening under high light	The ciliate *Mesodinium rubrum* produces mycosporine-like amino acids and their production is stimulated by light[80].
Adjusting kleptoplast density depending on light intensity/digesting dysfunctional kleptoplast	In general, kleptoplasts that lack control and repair mechanisms from the kleptokaryon or host nucleus exhibit a shorter lifespan until they are digested, whereas those with control and repair mechanisms exhibit a longer lifespan (see the main text.).
	Kleptoplasts are more rapidly turned over under higher intensity of light in the ciliates *Strombidium*[70] and *M. rubrum*[122], as well as in the dinoflagellate *Dinophysis*[86]. In several species of benthic foraminifera that acquire kleptoplasts from diatom prey, the digestion of kleptoplasts occurs earlier in light conditions compared to dark conditions, and earlier under high light intensity compared to low light intensity[71,72].
	The dinoflagellate *Dinophysis*, which obtain the kleptoplast from the ciliate *Mesodinium* prey, contains more and larger kleptoplasts when grown under low light compared to cells grown under high light[85].
	In the dinoflagellate *Nusuttodinium aeruginosum*, which obtains the kleptoplast and kleptokaryon from the cryptomonad *Chroomonas* sp. prey, cells that have lost the kleptokaryon and, therefore, the ability to repair damaged kleptoplasts, soon initiate the digestion of the kleptoplasts[82].
Maintenance and regulation of the photosynthetic apparatus by the kleptokaryon or host genome	In the ciliate *M. rubrum*, the kleptokaryon maintains, grows and photoacclimates the kleptoplast[77,80].
	In the dinoflagellate *N. aeruginosum*, the kleptokaryon maintains and grows the kleptoplast[81].
	In the dinoflagellate *Dinophysis*, the nucleus-encoded kleptoplast-targeted proteins maintains and grow the kleptoplast[87,88]. However, the photosynthetic apparatus cannot photoacclimate[85].
	In the Ross Sea dinoflagellate, which obtain the kleptoplast from the haptophyte *Phaeocystis antarctica*, the nucleus-encoded kleptoplast-targeted proteins maintains the kleptoplast[90]. However, the kleptoplast does not grow[89].

Supplementary Table 1. Mechanisms to mitigate photosynthetic oxidative stress evolved in eukaryotes other than ROS scavenging (*continued*).

Mechanisms	Notes
**(Photosymbiosis)**	
Host movement depending on the light intensity	Several species of benthic foraminifera exhibit negative phototaxis; moving into a shaded environment when exposed to high light. This movement does not occur in the presence of the photosynthetic inhibitor DCMU[111].
	The ciliates *Euplotes daidaleos* and *Paramecium bursaria* exhibit step-up (out-of-the-light) and step-down (into-the-light) photophobic responses. The step-down reaction is dependent on the presence of endosymbiotic *Chlorella*. On the other hand, the step-up reaction is an intrinsic response of the ciliate itself, but it is enhanced by the presence of endosymbiotic *Chlorella*[106].
Relocating endosymbionts depending on the light intensity	The benthic foraminifera *Marginopora vertebralis* relocates the symbiont, the dinoflagellate *Symbiodinium*, deeper into the cavities within its calcium carbonate test using actin upon high light exposure. This movement does not occur in the presence of the photosynthetic inhibitor DCMU[112].
	The ciliate *P. bursaria* aggregates symbiotic *Chlorella*, to shade both the host and the Chlorella cells, when exposed to high light. In low light conditions, it evenly distributes the *Chlorella* cells, maximizing light uptake[105].
Shading/sunscreening under high light	Several species of benthic foraminifera calcify denser and thicker shells under high light[109,110].
Adjusting endosymbiont density/digesting or expelling dysfunctional endosymbionts	The benthic foraminifera *Operculina ammonoides* changes the number of the diatom symbionts in accordance with changes in light quantity and quality[107]. In the benthic foraminifera *Amphistegina gibbosa*, high intensity of light or UV leads to bleaching. The bleaching results from digestion of the diatom endosymbionts[108].
	In the ciliate *P. bursaria*, the number of *Chlorella* endosymbionts per cell reaches its peak at moderate light levels, while it decreases under high light conditions[101]. When protein synthesis in the endosymbiotic Chlorella is inhibited in the light, *P. bursaria* digests the symbionts. This digestion does not occur in the dark or in the presence of the photosynthetic inhibitor DCMU in the light[102]. *P. bursaria* reduces the number of *Chlorella* endosymbionts per cell when the production of ROS by the endosymbiont is experimentally increased by high light exposure or the application of methylviologen[103].

Supplementary Table 1. Mechanisms to mitigate photosynthetic oxidative stress evolved in eukaryotes other than ROS scavenging (*continued*).

Mechanisms	Notes
**(Feeding on algae)**	
Digesting algal prey faster in the light than in the dark	Several species of ciliates, heterotrophic dinoflagellates, and *Naegleria* sp. (belonging to Discoba) exhibit faster digestion of algal prey under light conditions compared to dark conditions[13,114,115]. However, when *Naegleria* sp. is exposed to high light, it initially slows down the uptake of algal prey until it becomes acclimated to the new light condition[13]. These differences between light and dark are observed when predators feed on photosynthetic prey but not non-photosynthetic prey[13,114].
Shading/sunscreening under high light	When *Acanthamoeba* sp., *Vannella* sp. (both belonging to Amoebozoa), and *Naegleria* sp. are transferred from a dark environment to light, genes involved in carotenoid synthesis are upregulated. However, it has not been confirmed whether the production of carotenoids actually occurs[13].

Ref 122: Johnson, M. D. & Stoecker, D. K. Role of feeding in growth and photophysiology of *Myrionecta rubra*. *Aquat. Microb. Ecol*. **39**, 303–312 (2005).
